# Quantitative Synthesis of Microbe‐Driven Acclimation and Adaptation in Wild Vertebrates

**DOI:** 10.1111/eva.70025

**Published:** 2024-10-09

**Authors:** Garazi Martin Bideguren, Orly Razgour, Antton Alberdi

**Affiliations:** ^1^ Center for Evolutionary Hologenomics, Globe Institute University of Copenhagen Copenhagen Denmark; ^2^ Biosciences University of Exeter, Streatham Campus Exeter UK

**Keywords:** adaptation, microbiota, systematic review, wildlife management

## Abstract

Microorganisms associated with animals harbour a unique set of functional traits pivotal for the normal functioning of their hosts. This realisation has led researchers to hypothesise that animal‐associated microbial communities may boost the capacity of their hosts to acclimatise and adapt to environmental changes, two eco‐evolutionary processes with significant applied relevance. Aiming to assess the importance of microorganisms for wild vertebrate conservation, we conducted a quantitative systematic review to evaluate the scientific evidence for the contribution of gut microorganisms to the acclimation and adaptation capacity of wild vertebrate hosts. After screening 1974 publications, we scrutinised the 109 studies that met the inclusion criteria based on 10 metrics encompassing study design, methodology and reproducibility. We found that the studies published so far were not able to resolve the contribution of gut microorganisms due to insufficient study design and research methods for addressing the hypothesis. Our findings underscore the limited application to date of microbiome knowledge in vertebrate conservation and management, highlighting the need for a paradigm shift in research approaches. Considering these results, we advocate for a shift from observational studies to experimental manipulations, where fitness or related indicators are measured, coupled with an update in molecular techniques used to analyse microbial functions. In addition, closer collaboration with conservation managers and practitioners from the inception of the project is needed to encourage meaningful application of microbiome knowledge in adaptive wildlife conservation management.

Microorganisms associated with animals harbour a unique set of functional traits, which is increasingly recognised as pivotal for the normal functioning of their hosts (McFall‐Ngai et al. 2013). Nutrition, thermoregulation and behaviour are some of the biological processes of animals that are affected by microbial communities and their activity (Lee and Hase 2014). As microbial community compositions vary rapidly, microbial functions can change at a much faster rate than those encoded in host genomes (Alberdi et al. 2016). These observations led many researchers to set out the so‐called microbe‐driven adaptation hypothesis, whereby animal‐associated microbial communities would act as boosters of acclimation and adaptation capacity of their hosts through fast changes in the capacity to assimilate novel nutrients or to detoxify pernicious metabolites, for instance (Alberdi et al. 2016; Henry et al. 2021; Moeller and Sanders 2020; Rosenberg and Zilber‐Rosenberg 2022; Voolstra and Ziegler 2020). Microbial contributions to animal adaptation may be particularly relevant in vertebrates, as their longer generation times and fewer offspring slow the pace of genomic adaptation compared to other animals (Alberdi et al. 2016).

Measuring the dimensions of this contribution has significant applied relevance, as it can help us to better understand species' responses to anthropic pressures, such as land‐use or climate change (Dillard et al. 2022). If microorganisms act as buffers of environmental pressures (Henry et al. 2021), animals might be able to cope with changing conditions better than expected from their intrinsic genomic features (Alberdi et al. 2022). Microorganisms could also confer animals with the capacity to exploit novel resources under the new environmental conditions (Kohl et al. 2014). Ultimately, this knowledge could guide conservation and management strategies for threatened species (Zhu and Wang 2023), through implementing microbiome manipulation strategies in captive breeding programs (Trevelline et al. 2019) or identifying threatened populations through honing species distribution predictions (Razgour et al. 2019). However, testing the microbe‐driven hypothesis is not a trivial endeavour, because microbiota variation alone is not a synonym for adaptive advantage. Further analyses, such as microbiota transplants and host fitness measurements, are essential to establish causal relationships (Koh and Bäckhed 2020), and detailed functional analyses may be required to understand the underlying biological process (Koziol et al. 2023). Whether and how such strategies have been so far implemented by researchers has not been assessed yet.

We conducted a quantitative review of the literature to critically evaluate the effort so far conducted to elucidate the role of intestinal microorganisms' in vertebrate adaptation and assess the implications for vertebrate conservation. The studies that met our inclusion criteria were systematically analysed for 10 performance criteria related to three domains: experimental design, methodological resolution and reproducibility. Each criterion was quantitatively assessed to generate domain‐specific and overall performance scores by setting the weight of each criterion based on the judgement of independent experts. Based on the results of our quantitative assessment, we highlight the limitations of the research so far conducted and suggest methodological alternatives to improve the value of host‐associated microbiome research for animal conservation and management.

## 1 Methods

### 1.1 Literature Search

On the 1st of June 2023, we searched the Scopus (Elsevier) and Web of Science (Clarivate) databases for articles published since 2016, when the microbe‐driven adaptation hypothesis applied to vertebrates was published (Alberdi et al. 2016). The following string was used for the search: (wild*) AND (animal*) AND (adapt*) AND (“gut microbiota” OR “gut microbiome” OR “intestinal microbiota” OR “intestinal microbiome” OR “GUT microbiota” OR “GUT microbiome”). Publications were then filtered to retain only manuscripts classified as “Article”, “Letter”, “Article in Press”, “Note”, “Short Survey”, “Reprint” or “Article; Early Access.” The studies obtained after the systematic search and the initial filtering were manually screened to analyse whether they met the following inclusion criteria: the study (i) analyses the gut microbiota, (ii) revolves around or touches upon environmental adaptations, (iii) is focused on wild vertebrates and (iv) is based on empirical data. The studies retained after the second filtering were scrutinised to assign performance scores as explained below (see the PRISMA diagram in Figure S1).

### 1.2 Scoring System

We quantified the performance of studies in terms of addressing the microbial adaptive contribution hypothesis, by considering 10 criteria clustered into three domains: experimental design, methodological resolution and reproducibility. The criteria were chosen to encompass the diverse features of a research study. Each criterion included various descriptors, representing a spectrum of options from the weakest to the strongest approaches for addressing the adaptive hypothesis (Figure 2, Table S1).

Experimental design encompassed four criteria: (i) ‘Experimental approach’ assigned increasing scores from descriptive/observational, which only enable correlations, to experimental manipulation approaches that enable the establishment of causation (Meijnikman et al. 2018). (ii) ‘Analysis approach’ assessed whether the study attempted to ascertain the mechanism of microbial action on host biology, rather than only describing a correlative pattern. (iii) ‘Hypothesis testing’ assessed whether the microbe‐driven adaptation was explicitly addressed in the study. (iv) The ‘Sample size’ criterion assigned higher scores to larger sample sizes, due to their increased capacity to capture microbiome variability more comprehensively (Kers and Saccenti 2021). A simple metric was chosen due to the variability in microbial community differences across hosts and the challenges in defining and matching relevant group sizes in diverse experimental designs. Methodological resolution grouped five criteria: (v) ‘Fitness measurement’ evaluated whether the study quantified host fitness, a crucial aspect for assessing whether microbial differences exerted a significant impact on the host. (vi) ‘Host resolution’ criterion assessed the resolution of the host‐microbiota analysis, assigning higher scores to studies that analysed hosts at the individual level, where host‐microbiota interactions occur, rather than in groups. (vii) ‘Functional response’ assigned higher scores based on the capability of the methodologies (e.g., genome‐resolved metagenomics higher capability than functionality inferred from taxonomy) used to provide detailed information on biological functioning (Srivastava et al. 2019). (viii) ‘Molecular technique’ allocated higher scores to studies using methodologies that capture a broader range of molecular information (e.g., multi‐omics vs. targeted sequencing). (ix) ‘Compositional variability resolution’ evaluated the resolution of microbiome comparison analyses, assigning higher scores to methods that account for functional microbiome traits compared to those that do not (e.g., richness‐based vs. functional diversity‐based analyses). Finally, within the Reproducibility domain, (x) ‘Data availability’ allocated scores based on the extent of resources provided to reproduce the experiment.

Each criterion included between 2 and 6 incremental options, which spanned quantitative scores between two options (0 or 1) and six options (0, 0.2, 0.4, 0.6, 0.8 and 1), as indicated in the (Table S1). The lowest value represents the weakest and the highest value the most robust approach to address the microbial adaptive contribution hypothesis. In the case of reproducibility, availability of raw data was set as a condition to obtain a positive score value. Each of the 10 analysed criteria was given a different weight to obtain the overall performance scores through weighted average calculation. To minimise subjectivity, we requested eight independent experts to assign relevance values to each of the 10 criteria to address the hypothesis of interest. The weights employed in the final analysis were derived by averaging the values provided by the experts (Table S2). For example, the relative weight assigned to the fitness measurement criterion was twofold larger than the weight assigned to the criterion that evaluated the resolution of microbiome variation measurements. We then assessed the studies in terms of result interpretation, considering whether the study explicitly addressed the adaptive hypothesis as its foundation or not.

### 1.3 Statistical Analyses

We used linear models to explore the quantitative relationships between the studied criteria, domains and overall performance scores, against publication time (calendar years). We employed Kruskal–Wallis test to analyse differences in performance score across geography (continents) and taxa (vertebrate classes, with Actinopterygii and Dipnoi merged within “fish”), followed by post hoc Dunn test with the Benjamini–Hochberg adjustment procedure for pairwise comparisons. All analyses and visualisations were produced in the R statistical software v.4.2.2, using vegan v.2.6–4, FSA v.0.9.5 and ggplot2 v.3.5.0 packages respectively. Final composite figure layouts were edited in Adobe Illustrator 2024.

## 2 Results

**FIGURE 1:**
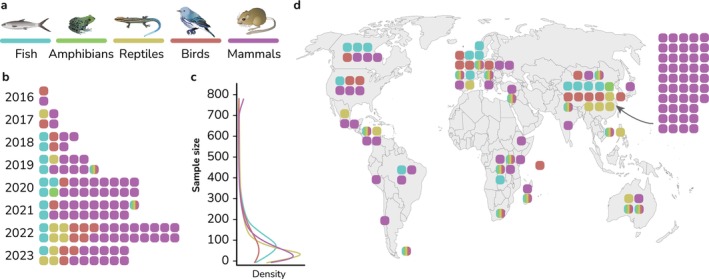
Overview of the analysed studies. (a) Vertebrate groups included in the analysed studies. (b) Studies divided by year and coloured by the vertebrate group used for the research. Rainbow boxes indicate studies encompassing multiple vertebrate classes. Note that only the first half of the year is included in 2023. (c) Density curve of sample sizes of each vertebrate group included in the analysed studies. Note that amphibians are not included due to small sample size. Multitaxon studies are also excluded. (d) Approximate geographic distribution of the origin of the studied animals. Note that some studies analysed animals from various locations.

Our systematic search of the Scopus and Web of Science publication databases yielded 1974 articles that corresponded to our keywords. After manually screening these articles, we retained with a final set of 109 studies that met our inclusion criteria. These studies were dominated by research on mammals, followed by birds, fish, reptiles and amphibians (Figure 1a,b). The number of analysed animal individuals ranged between 2 and 758, with an average number of 77.9 ± 105.8 individuals per study (Figure 1c). Geographic distribution spanned six continents, with a clear dominance of Asia, especially China, followed by Europe (Figure 1d). Major geographic gaps were identified in North Africa, Central Asia and Melanesia.

**FIGURE 2:**
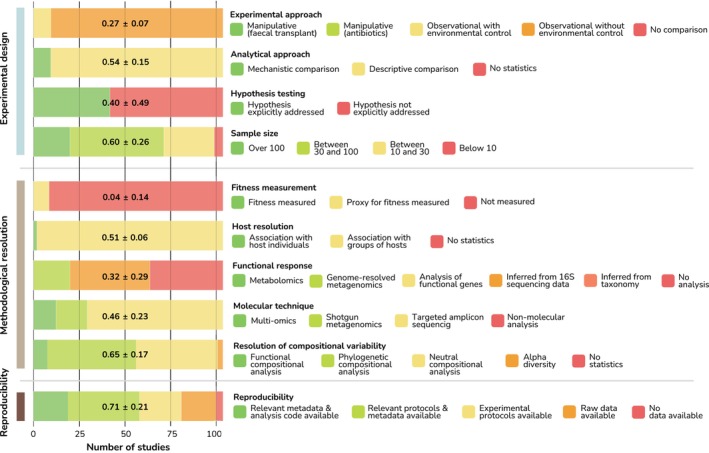
Performance scores of assessed criteria. Criteria and their corresponding descriptor divided in colours in each of the domains, together with the overall performance scores of the criteria.

**FIGURE 3:**
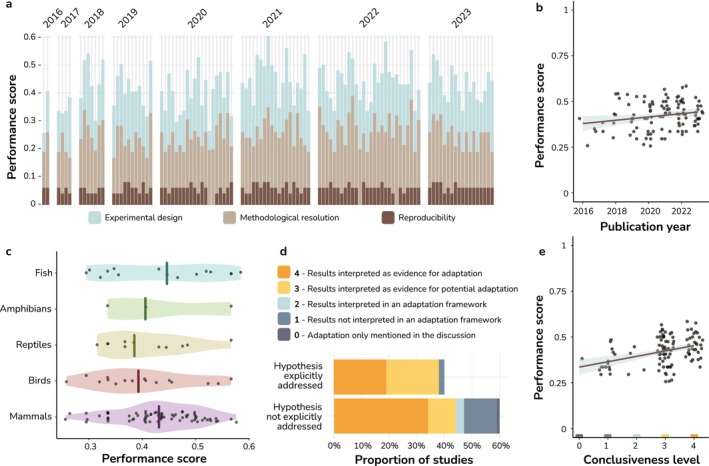
Performance scores of the analysed studies. (a) Total performance scores of each study with the relative contribution of each domain indicated by a different colour. (b) Linear regression between the performance score and the publication year. (c) Average performance scores of studies clustered by the taxonomic group. (d) Result interpretation of the studies with regards to adaptation, split between studies that explicitly addressed the increased adaptability provided by microbiomes and those that did not. (e) Linear regression between the performance score and the result interpretation in the studies. Result interpretation levels refer to the legend in (d).

Performance scores differed across criteria. The resolution of compositional variability, sample size and reproducibility yielded the highest scores, with average performance values over 0.6 out of 1. In contrast, fitness measurement was the criterion with the lowest score, with only 9 out of the 109 studies having measured proxies for host fitness (Figure 2). The overall performance score was 0.42 ± 0.07, with minimum and maximum values ranging between 0.25 and 0.6 (Figure 3a). Average scores increased slightly, yet significantly, over time by 0.1% per year (LM; *β* = 0.001 points/year, *F* = 2.687, DF_*nom/den*_ = 1/107, *p* = 0.008, Figure 3b), driven by non‐significant trends of temporal increases in the three domains analysed (Table S3, Figure S2). There were no differences in total scores among continents or taxa (Figure 3c, Table S4). Among studies with an explicit hypothesis regarding microbial contributions to animal adaptation (comprising 40% of the total analysed, i.e. 44 studies), nearly all interpreted their results as evidence or potential evidence of adaptation (Figure 3d). In the case of studies lacking an explicit hypothesis about microbial contributions to animal adaptation (60%, *n* = 65), most researchers still interpreted their findings as indicative of potential or clear signs of adaptation facilitated by gut microbes. Finally, we observed a positive correlation between the result interpretation reported by the authors and the performance scores we assessed (LM; *β* = 0.029 points/year, *F* = 4.232, DF_*nom/den*_ = 1/107, *p <* 0.001, Figure 3e), although average performance scores remained below 0.5 in the group of studies that interpreted findings as evidence for adaptation.

## 3 Discussion

Our systematic review represents the first quantitative evaluation of the available evidence pertaining to the microbial influence on the acclimation and adaptation capacity of vertebrates. The significant volume of publications meeting our inclusion criteria underscores the focus of the researcher community on the hypothesis, suggesting that gut microorganisms may contribute to the acclimation and adaptation capacity of their vertebrate hosts. However, our systematic analysis of the literature revealed that the field has yet to align with ideal research practices to address this complex hypothesis. In the ensuing discussion, we address the primary limitations highlighted by our analyses and propose methodological alternatives to enhance the relevance of host‐associated microbiome research in the context of animal conservation and management.

### 3.1 Study Design

One notable limitation we detected in experimental designs was the very limited control exerted over environmental conditions and gut microbiomes. Whereas only a few studies controlled for environmental conditions, no study manipulated gut microbiomes, an essential factor for establishing causal relationships between microbial communities and host responses (Koh and Bäckhed 2020). Inoculation of germ‐free animals with microbial communities is feasible only with model species, due to the complex infrastructure required for creating and sustaining gnotobiotic animals (Al‐Asmakh and Zadjali 2015). When working with wild systems, more practical alternatives include treating animals with antibiotics to deplete any microbial activity that could contribute to acclimation (Knutie et al. 2017) or conducting faecal microbiota transplants between animals adapted to different conditions (Guo et al. 2020). Incorporating these experimental manipulations would help mitigate the inherent constraints of correlative approaches and provide a pathway toward elucidating causal relationships between microbial communities and adaptive capacity of animals (Gheorghe et al. 2021). In addition, assessing the success of microbiota transplants would also contribute to optimising microbiota manipulation strategies that can be implemented in future conservation actions (Guo et al. 2020).

Similarly, the great majority of studies did not explicitly measure fitness or related proxies, with only 5% of the studies incorporating host responses in terms of animal physiology, reproduction or survival. Absence of fitness measurements hinders the ability to quantify the extent of the microbiota's adaptive contribution to their hosts, rendering any conclusions speculative (Suzuki 2017). It is worth noting that directly measuring fitness through reproductive success or survival metrics in vertebrates is challenging due to complex experimental setups and study durations (Barnett et al. 2015). Additionally, ethical considerations and related legislation may limit the use of survival as a fitness indicator (Verderio et al. 2023). Alternatives, such as assessing physiological parameters (e.g., calorimetry and respirometry) or behavioural metrics, can serve as valuable proxies for fitness (Dichiera, Khursigara, and Esbaugh 2021).

### 3.2 Molecular Methodologies

Maximum scores of the molecular approaches employed increased steadily over time, indicating that more studies delved into the functional interactions between microbes and hosts. Nonetheless, average scores assigned to the methodology exhibited little variation over the eight screened years, due to the ubiquitous reliance on 16S rRNA amplicon sequencing in the majority of studies. Although cost‐effective for taxonomic characterisation of microbiomes, this approach offers limited insights into the functional role of microbes in host interactions (Bharti and Grimm 2021), which ultimately impedes our ability to unravel mechanistic processes. Studying the functional contributions of microorganisms can provide deeper insights into the mechanisms through which microorganisms enhance vertebrates' acclimation capacity (Koziol et al. 2023) and therefore inform strategies to best modulate microbiomes (Shetty et al. 2017). This can be achieved through genome‐resolved metagenomics, which allows for the near‐complete reconstruction of bacterial genomes from faecal samples (Quince et al. 2017), which can then be functionally annotated to gain insights into their metabolic capacities (Shaffer et al. 2020). The incorporation of RNAseq transcriptomics data not only provides information on functional capacities but also measures the actual functional activity of microorganisms by identifying genes that are differentially expressed (up‐ or down‐regulated) under different conditions.

### 3.3 Result Interpretation

Most studies analysed in this review claimed evidence or potential evidence for microbe‐driven vertebrate adaptation. The relationship between the performance scores we measured and how researchers interpreted their findings indicates that authors adjusted their claims to the robustness of the generated evidence to a certain degree. However, the lack of microbiota manipulation and host fitness measurements in most studies hindered researchers from generating conclusive evidence (Koh and Bäckhed 2020). This missing information also obstructed our ability to conduct quantitative meta‐analyses on the effect sizes of microbe‐driven host adaptation. The relatively low performance scores measured in our systematic review further suggest a tendency among researchers to make more assertive claims than probably warranted by their results. In consequence, we advocate for a more cautious interpretation of results, to prevent allocating resources to ineffective microbiome applications that are based on weak or inconclusive evidence.

### 3.4 Impact on Evolutionary Applications

Investigating the impact of microbes across various stages of vertebrate evolution is a key inquiry in the field of evolutionary biology. A wealth of evidence indicates that patterns of diversity and compositional variation of the gut microbiota align with a range of eco‐evolutionary traits in vertebrates, including dietary niche and ecophysiology (Ley et al. 2008; Song et al. 2020). Nevertheless, these patterns do not elucidate the precise evolutionary processes by which microorganisms may influence the evolutionary trajectories of vertebrate hosts. This underscores the need for more mechanistic studies that delve into direct measurements of physiological parameters and fitness effects in the context of variations in the microbiome (Fontaine and Kohl 2020; Kohl and Carey 2016). Gut microorganisms have been hypothesised to act as boosters of vertebrate acclimation and adaptation capacity (Alberdi et al. 2016). However, our systematic review indicated that the scientific community has not yet generated enough evidence to address the question of whether gut microorganisms confer wild vertebrates with enhanced acclimation and adaptation capacity, hindered by limitations in the experimental and analytical approaches employed.

The current restricted knowledge in this area impedes our ability to assess the relevance of microorganisms as pivotal factors influencing animal conservation, as well as their potential as valuable assets in conservation practices. For instance, the quantification of microbiome effects on host heat tolerance and susceptibility to warming across vertebrates would empower us to formulate more accurate predictions of responses to climate change (Fontaine and Kohl 2023; Razgour et al. 2019). In addition, ascertaining whether animals from different populations harbour adapted microbiomes with key traits for host fitness could shed light on the feasibility of transferring microbial communities to vulnerable populations that stand to benefit from these microorganisms (West et al. 2019). Similarly, determining whether pre‐conditioning captivity‐bred animals with wild‐like microbiotas enhances their fitness would be crucial information for the implementation of effective reintroduction programmes (Diaz and Reese 2021; Trevelline et al. 2019).

The limited understanding of the relevance of microorganisms for animal conservation likely mirrors the complexity of the research required to tackle this scientific question. Addressing the microbe‐driven acclimation and adaptability hypothesis requires a combination of expertise that includes experimental study design, access to appropriate study systems and experimental facilities, capacity to measure fitness or proxies for fitness and proficiency in complex molecular data generation and analysis. This is a mix of competencies that calls for collaborative research. If we are to address this question, a shift from observational to experimental manipulation approaches is imperative, coupled with the modernisation of molecular techniques used to characterise microbiomes and host responses, leveraging the potential of multi‐omic methodologies. Moreover, involving conservation managers and practitioners from the inception of the study is essential for addressing questions relevant for wildlife conservation and ensuring the application of the results. Only then will we be able to quantify the intensity and breadth of the suggested contribution of gut microorganisms to vertebrate acclimation and adaptation capacity and effectively apply microbiome knowledge to improve animal conservation and adaptive management actions.

## Conflicts of Interest

The authors declare no conflicts of interest.

## Supporting information


**Data S1.** Supporting Information.

## Data Availability

All data are available at https://github.com/alberdilab/microbiota_adaptation_review (data folder). A release of the repository is frozen in Zenodo with https://doi.org/10.5281/zenodo.10081288.
