# Supplementary material for: Quantitative Synthesis of Microbe‐Driven Acclimation and Adaptation in Wild Vertebrates
Source: Evol Appl. 2024 Oct 9;17(10):e70025. doi: 10.1111/eva.70025 (PMC11464772; doi:10.1111/eva.70025)
Supplement: Supplementary file 1 — Data S1. Supporting Information. [file EVA-17-e70025-s001.docx]

**Supplementary material**

Quantitative synthesis of microbe-driven acclimation and adaptation in wild vertebrates

Garazi Martin Bideguren^1^, Orly Razgour^2^, Antton Alberdi^1^

^1^Center for Evolutionary Hologenomics, Globe Institute, University of Copenhagen, 1353 Copenhagen, Denmark.

^2^Biosciences, University of Exeter, Streatham Campus, Exeter EX4 4PS, UK.

**Correspondence:** Antton Alberdi, [antton.alberdi@sund.ku.dk](mailto:antton.alberdi@sund.ku.dk)

**Table of contents**

[Supplementary tables 2](#_Toc178140037)

[Table S1 2](#_Toc178140038)

[Table S2 3](#_Toc178140039)

[Table S3 4](#_Toc178140040)

[Table S4 4](#_Toc178140041)

[Supplementary figures 5](#_Toc178140042)

[Figure S1 5](#_Toc178140043)

[Figure S2 6](#_Toc178140044)

#

# Supplementary tables

## Table S1

Description of the scoring criteria and the value assigned to each ordinal category.

| **Domain** | **Criterion** | **Description** | **Score** |
| --- | --- | --- | --- |
| Experimental design | Experimental approach | No comparison | 0 |
|  |  | Observational without environmental control | 0.25 |
|  |  | Observational with environmental control | 0.5 |
|  |  | Manipulative (antibiotics) | 0.75 |
|  |  | Manipulative (faecal tranplant) | 1 |
|  | Analysis approach | No statistics employed | 0 |
|  |  | Descriptive comparison | 0.5 |
|  |  | Mechanistic comparison | 1 |
|  | Hypothesis testing | Microbe-driven adaptation hypothesis not explicitly addressed | 0 |
|  |  | Microbe-driven adaptation hypothesis explicitly addressed | 1 |
|  | Sample size | Below 10 | 0 |
|  |  | Between 10 and 30 | 0.33 |
|  |  | Between 30 and 100 | 0.66 |
|  |  | Over 100 | 1 |
| Methodological resolution | Fitness measurement | Fitness not measured | 0 |
|  |  | Proxy for fitness measured | 0.5 |
|  |  | Fitness measured | 1 |
|  | Host resolution | No statistics | 0 |
|  |  | Association with host groups | 0.5 |
|  |  | Association with host individuals | 1 |
|  | Functional response | No functional analysis | 0 |
|  |  | Inferred from taxonomy | 0.2 |
|  |  | Inferred from 16S | 0.4 |
|  |  | Analysis of f unctional genes | 0.6 |
|  |  | Genome-resolved metagenomics | 0.8 |
|  |  | Metabolomics | 1 |
|  | Molecular technique | No sequencing-based | 0 |
|  |  | Targeted sequencing | 0.33 |
|  |  | Shotgun metagenomics | 0.66 |
|  |  | Multi-omics | 1 |
|  | Compositional variability resolution | No statistics | 0 |
|  |  | Alpha diversity | 0.25 |
|  |  | Neutral compositional analysis that only consider relative abundances of microorganisms | 0.5 |
|  |  | Phylogenetic compositional analysis that accounts for the phylogenetic relationships among microorganisms | 0.75 |
|  |  | Functional compositional analysis that consider the functional traits of the microorganisms | 1 |
| Reproducibility | Data availability | No data available | 0 |
|  |  | Raw data available | 0.25 |
|  |  | Detailed experimental protocols | 0.5 |
|  |  | Origin of animals | 0.75 |
|  |  | Bioinformatics/statistics code | 1 |

## Table S2

Relative weight of each criterion given by each of the experts and the average value used for the analyses.

| **Criteria** | **Exp 1** | **Exp 2** | **Exp 3** | **Exp 4** | **Exp 5** | **Exp 6** | **Exp 7** | **Exp 8** | **Avg** |
| --- | --- | --- | --- | --- | --- | --- | --- | --- | --- |
| Experimental approach | 0.130 | 0.083 | 0.067 | 0.048 | 0.143 | 0.063 | 0.176 | 0.091 | **0.1** |
| Analysis approach | 0.087 | 0.083 | 0.067 | 0.095 | 0.095 | 0.125 | 0.059 | 0.091 | **0.09** |
| **Hypothesis testing** | 0.087 | 0.042 | 0.067 | 0.048 | 0.095 | 0.063 | 0.059 | 0.091 | **0.07** |
| Sample size | 0.130 | 0.125 | 0.133 | 0.143 | 0.095 | 0.063 | 0.118 | 0.136 | **0.12** |
| Fitness measurement | 0.130 | 0.125 | 0.200 | 0.143 | 0.143 | 0.188 | 0.176 | 0.091 | **0.15** |
| **Host resolution** | 0.130 | 0.125 | 0.133 | 0.095 | 0.095 | 0.063 | 0.118 | 0.136 | **0.11** |
| Functional response | 0.087 | 0.125 | 0.133 | 0.143 | 0.048 | 0.188 | 0.059 | 0.136 | **0.12** |
| Molecular technique | 0.043 | 0.083 | 0.067 | 0.095 | 0.095 | 0.063 | 0.059 | 0.045 | **0.07** |
| Compositional variability resolution | 0.130 | 0.083 | 0.067 | 0.095 | 0.095 | 0.125 | 0.059 | 0.136 | **0.1** |
| Data availability | 0.043 | 0.125 | 0.067 | 0.095 | 0.095 | 0.063 | 0.118 | 0.045 | **0.08** |
| **Total** | **1** | **1** | **1** | **1** | **1** | **1** | **1** | **1** | **1** |

#

## Table S3

Linear model results of the temporal trends of performance scores

| **Performance metric** | **Parameter estimate** | **DF***_nom/den_* | **t-value** | **p-value** |
| --- | --- | --- | --- | --- |
| Total ~ Year | 0.009 | 1/107 | 2.196 | 0.030 |
| Experimental design ~ Year | 0.012 | 1/107 | 1.641 | 0.104 |
| Methodological resolution ~ Year | 0.008 | 1/107 | 1.495 | 0.138 |
| Reproducibility ~ Year | 0.001 | 1/107 | 0.034 | 0.973 |

## Table S4

Kruskal-Wallis test of the difference of performance scores across continents and taxa.

| **Comparison** | **chi-squared** | **DF** | **p-value** |
| --- | --- | --- | --- |
| Total ~ Continent | 5.3242 | 5 | 0.3776 |
| Total ~ Taxa | 2.8549 | 4 | 0.5824 |

# Supplementary figures

## Figure S1


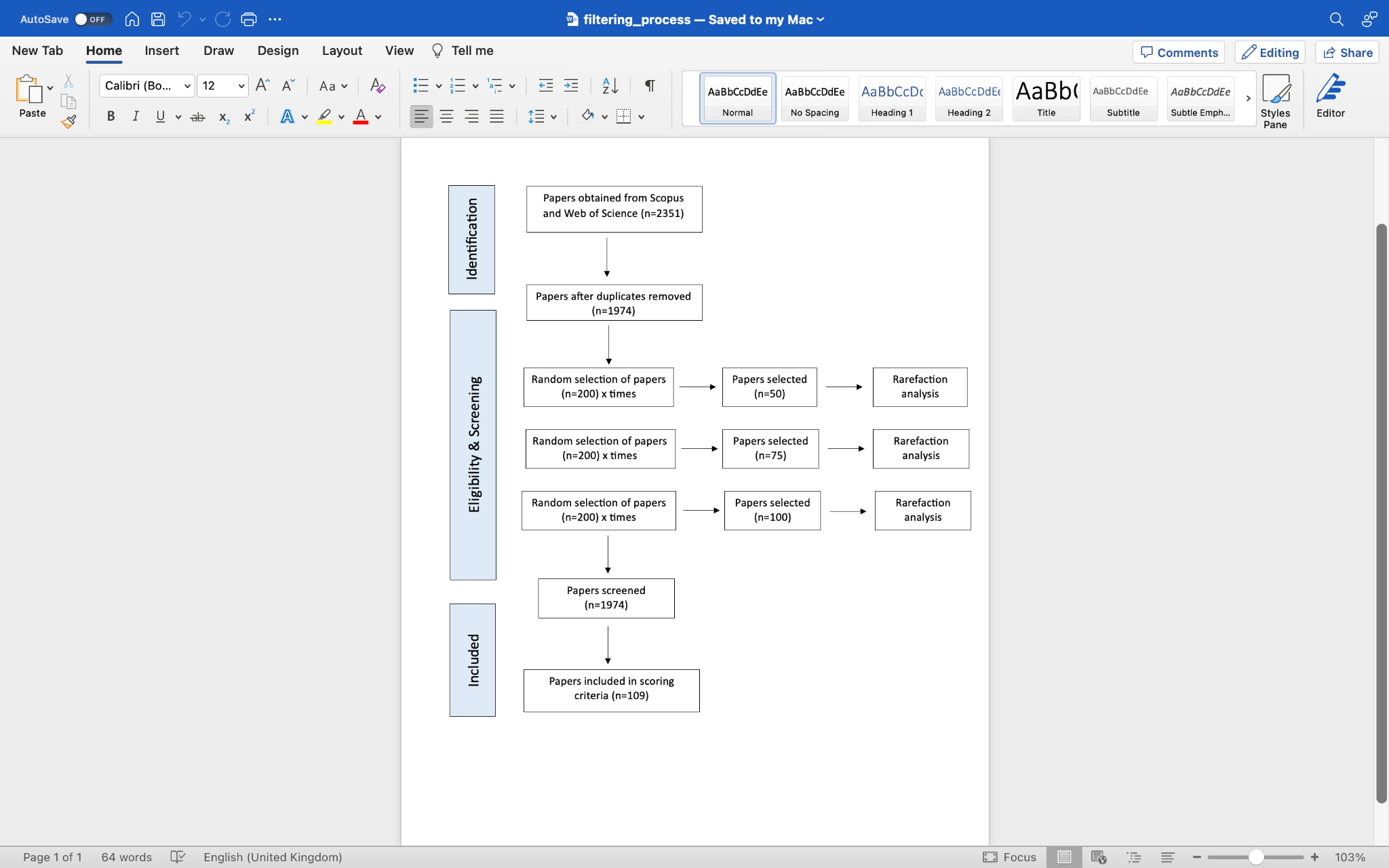


PRISMA chart of the filtering process.

## Figure S2


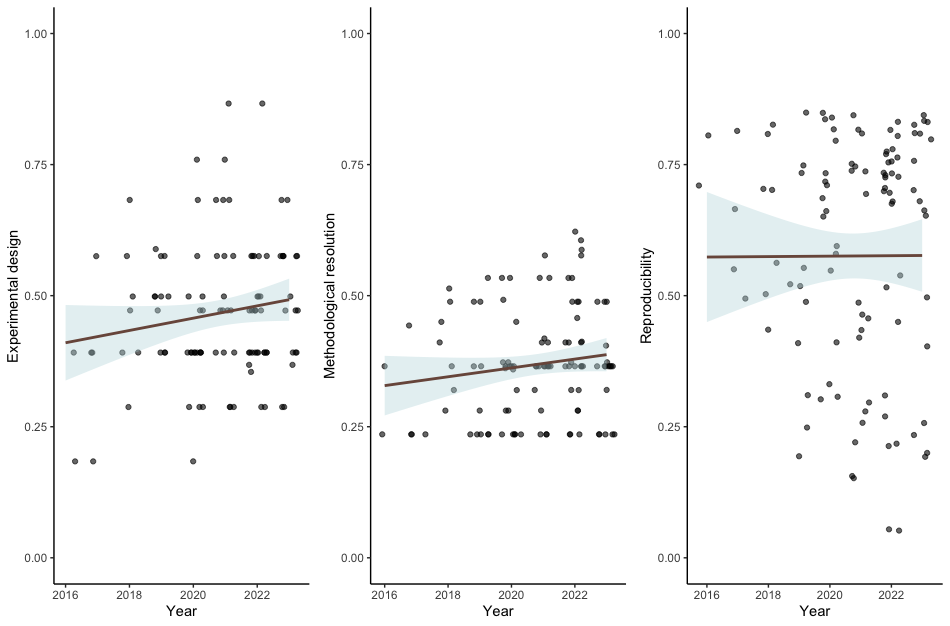


Temporal trends of performance scores in each of the domains.
